# Unravelling the impact of frontal lobe impairment for social dysfunction in myotonic dystrophy type 1

**DOI:** 10.1093/braincomms/fcac111

**Published:** 2022-05-17

**Authors:** Alexandre Morin, Aurelie Funkiewiez, Alexandre Routier, Raphael Le Bouc, Nicolas Borderies, Damien Galanaud, Richard Levy, Mathias Pessiglione, Bruno Dubois, Bruno Eymard, Claire-Cecile Michon, Nathalie Angeard, Anthony Behin, Pascal Laforet, Tanya Stojkovic, Carole Azuar

**Affiliations:** 1 Institut du Cerveau et de la Moelle épinière (ICM), UMRS 975, ICM-INSERM 1127, 75013 Paris, France; 2 Service de Neurologie, CHU Rouen, Centre National de Référence Maladie d’Alzheimer du sujet jeune, 76000 Rouen, France; 3 Département de Neurologie, Institut de la Mémoire et de la Maladie d’Alzheimer, Centre National Démences Rares, Hôpital Pitié-Salpêtrière, APHP, 75013 Paris, France; 4 Urgences cérébro-vasculaires, Hôpital de la Pitié-Salpêtrière, AP-HP, 75013 Paris, France; 5 Service de Neuroradiologie, Hôpital Pitié-Salpêtrière, APHP, 75013 Paris, France; 6 Unité de Neuro-Psychiatrie Comportementale (IHU), Hôpital de la Pitié-Salpêtrière, AP-HP, 75013 Paris, France; 7 Centre de référence des maladies neuromusculaires Nord/Est/Ile de France, Institut de Myologie, Hospital Pitié-Salpêtrière, APHP, 75013 Paris, France; 8 Centre de référence des maladies neuromusculaires Nord/Est/Ile de France, Institut de Myologie, Hospital Raymond Poincaré, APHP, 92380 Garches, France; 9 U1129, Paris Descartes University, Sorbonne Paris Cité, Paris, France; 10 Institut de Myologie, Groupe Hospitalier Pitié-Salpêtrière, APHP, Paris, France

**Keywords:** myotonic dystrophy type 1, behavioural neurology, neuropsychology, computational neuroimaging, social cognition

## Abstract

Myotonic dystrophy type 1 is an autosomal dominant multisystemic disorder affecting muscular and extra muscular systems, including the central nervous system. Cerebral involvement in myotonic dystrophy type 1 is associated with subtle cognitive and behavioural disorders, of major impact on socio-professional adaptation. The social dysfunction and its potential relation to frontal lobe neuropsychology remain under-evaluated in this pathology. The neuroanatomical network underpinning that disorder is yet to disentangle. Twenty-eight myotonic dystrophy type 1 adult patients (mean age: 46 years old) and 18 age and sex-matched healthy controls were included in the study. All patients performed an exhaustive neuropsychological assessment with a specific focus on frontal lobe neuropsychology (motivation, social cognition and executive functions). Among them, 18 myotonic dystrophy type 1 patients and 18 healthy controls had a brain MRI with T_1_ and T_2_ Flair sequences. Grey matter segmentation, Voxel-based morphometry and cortical thickness estimation were performed with Statistical Parametric Mapping Software SPM12 and Freesurfer software. Furthermore, T_2_ white matter lesions and subcortical structures were segmented with Automated Volumetry Software. Most patients showed significant impairment in executive frontal functions (auditory working memory, inhibition, contextualization and mental flexibility). Patients showed only minor difficulties in social cognition tests mostly in cognitive Theory of Mind, but with relative sparing of affective Theory of Mind and emotion recognition. Neuroimaging analysis revealed atrophy mostly in the parahippocampal and hippocampal regions and to a lesser extent in basal ganglia, regions involved in social navigation and mental flexibility, respectively. Social cognition scores were correlated with right parahippocampal gyrus atrophy. Social dysfunction in myotonic dystrophy type 1 might be a consequence of cognitive impairment regarding mental flexibility and social contextualization rather than a specific social cognition deficit such as emotion recognition. We suggest that both white matter lesions and grey matter disease could account for this social dysfunction, involving, in particular, the frontal-subcortical network and the hippocampal/arahippocampal regions, brain regions known, respectively, to integrate contextualization and social navigation.

## Teaser

Morin *et al*. report that social dysfunction in myotonic dystrophy type 1 results from a 2-fold mechanism: on one hand, frontal and basal ganglia loops lesion altering the executive functions, including cognitive Theory of Mind; on the other hand, hippocampus and parahippocampus lesions altering the social navigation and emotional contextualization.

## Graphical Abstract

**Graphical abstract:**
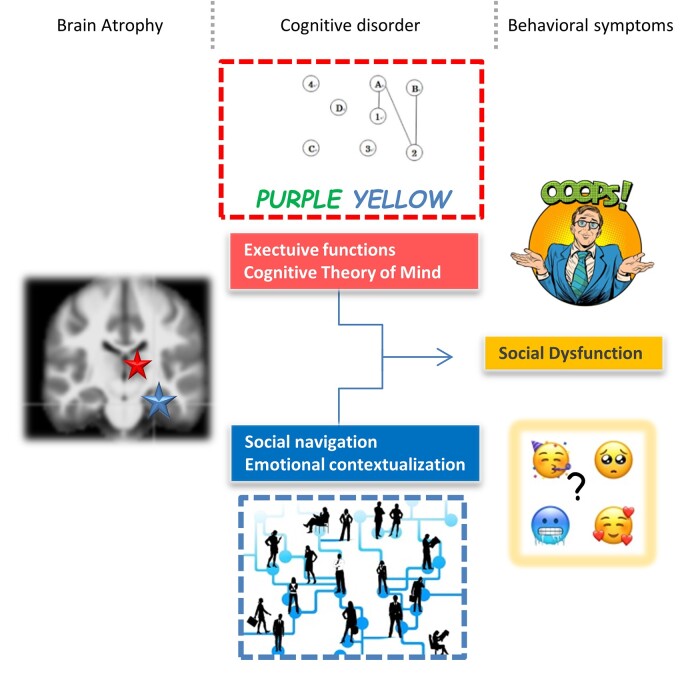

## Introduction

Myotonic dystrophy type 1 (DM1) is an autosomal dominant multisystemic disorder caused by a cytosine thymine guanine (CTG) repeat expansion, affecting both muscular and extra muscular systems including the central nervous system.^1–3^ Neuropathological studies suggest the presence of neurofibrillary tangles in DM1 brain, with tau isoform aggregation, mostly in the limbic system, the hippocampus and the entorhinal cortex,^4–6^ both key regions for human cognition.

Cognition has been a main concern in DM1 patients for the past 20 years, and there is a growing evidence of frontal lobe dysfunction in these patients, involving both the executive system and the social cognition network.

Indeed, global cognition in DM1 patients has been widely studied, revealing impairment in various functions such as memory (encoding and retrieval deficit), visuospatial (perception and construction) and attentional functions.^7–9^ Proper executive functions such as planning, mental flexibility, categorization, abstraction, rule elaboration, inhibition and contextualization have been specifically tested and are significantly impaired in these patients.^9,10^ Memory deficits (concerning encoding and retrieval processes), and visuospatial constructions deficits have been both related to this executive dysfunction.

Thus, executive dysfunction could be one of the core cognitive deficits in DM1 patients (possibly related to damages in the dorsolateral part of the frontal lobe and its cortico-striatal loops^11^).

Social dysfunction is also described in DM1patients,^12,13^ as a consequence of a social cognition disorder,^14,15^ but there is still a debate concerning its mechanism. Some studies made the assumption of a deficit of Theory of Mind (ToM), other studies of a deficit in emotion recognition or a lack of empathy.

Furthermore, some DM1 patients seem to suffer from a severe apathy or a lack of motivation impacting their quality of life.^12,13,16^ All these functions are underpinned by social cognition networks, namely the orbital part of the frontal lobe and its connexion to the temporal lobe^14,17–20^).

Thus through various cognition and behavioural disorder, there is a theoretical evidence of frontal lobe and its networks involvement in DM1 cognition deficit but the precise anatomo-clinical correlation is yet to disentangle.

Despite the growing literature on neuroimaging in DM1, only a few studies reported an association between neuropsychological impairment and brain localizations with controversial findings, especially concerning frontal lobe anatomy and neuropsychology.^21^ Various techniques have been implemented: MRI-based anatomical imaging measuring cortical atrophy,^22–24^ white matter (WM) tracts disruption,^17,25–28^ through metabolic imaging with Positron emission tomography–computed tomography^29^ and functional MRI.^30^

There is a need for new studies exploring DM1 cognition and behaviour along with its underpinning neuroanatomy, to better understand the social dysfunction of these patients. Here, we propose to study executive and socio-emotional functions in DM1 patients, with extensive neuropsychological tests, and we propose to correlate their cognitive and emotional pattern with brain anatomy, using various neurocomputational-imaging techniques.

## Materials and methods

Twenty-eight adult-onset DM1 patients, genetically confirmed, were recruited in Pitié-Salpêtrière Hospital, Paris, France, within the neuromuscular reference centre (Institut de Myologie).

Were excluded patients with:

- Developmental delay (in learning, walking or speech development) or repeated grade retention, suspects of a juvenile/childhood onset or congenital form of DM1, criteria assessed by patient and caregiver’s questionnaire and through medical history.
- Pacemaker (MRI contraindication).
- Other medical conditions implying cognitive disorders.

All subjects provided written informed consent. The local ethics committee of Pitié-Salpêtrière Hospital approved the study.

All patients performed an exhaustive neuropsychological assessment with a specific focus on cognitive and behavioural frontal functions. Twenty-two patients performed the apathy computerized task. We included 22 age and sex-matched controls recruited in Pitié Salpêtrière on a voluntary basis to perform the same motivation computerized task.

Eighteen out of the 28 DM1 patients performed a brain MRI with 3D T_1_ and T_2_ Flair sequences (MRI DM1 Group).

We included eighteen age and sex-matched healthy controls for the neuroimaging study (MRI Control Group). They were recruited in the same Imaging centre (Pitié Salpêtrière) on a voluntary basis.

### Neuropsychological tasks

The neuropsychological assessment included a screening test for deficits in global cognition (mini-mental state examination),^31^ verbal fluency with phonemic (words starting with P) and semantic cues (animals), respectively, literal and categorical fluency,^32^ verbal and spatial short-term memory (Digit Span forward and Spatial Span^33^), verbal long-term memory (five-word test^34^).

Executive functions evaluation included tests evaluating information processing, speed and mental flexibility (Trail Making Test A and B^35^), inhibition (Stroop Test^36^ and Hayling Test^37^), contextualization and rule elaboration (Wisconsin Sorting Cart Test^38^ and Brixton Test^37^). Social cognition tests included two subtests from the Social Emotion Assessment (SEA) Faux-Pas Test and Emotion recognition subtest.^39^ Apathy was evaluated with Starkstein Scale.^40^

An experienced neuropsychologist passed the tests in a quiet environment at the hospital site. Sixty to ninety minutes were required to fulfil the entire cognitive battery.

The patient’s performances were compared to normative published data for each test. A deviation score ranging below −1.66 standard deviation was considered as pathological.

### Apathy evaluation

The usual apathy evaluation scales rely on self or hetero-evaluation and only through questionnaires. Thus, those scales does not capture the various mechanisms leading to apathy (initiation, effort/reward rating, planification^41^).

Therefore, we performed a computerized battery evaluating various components of motivation (the subjective perception of effort cost and reward value, the willingness to exert effort for reward and choice impulsivity in inter-temporal choices) based on a paradigm designed by Pessiglione *et al*.^42^

### MRI

All brain MRIs included a 3D T_1_-weighted sequence and a FLAIR sequence with a spatial resolution of 1 × 1 × 1 mm^3^ and a magnetic field strength of 3 T. We applied the same MRI protocol for DM1 MRI patients and MRI controls.

### Neuroimaging analysis

#### Fully Automated Volumetry Software

Automated volumetry analyses were performed using the volBrain software (http://volBrain.upv.es), an online freely available academic brain image analysis tool.^43^

Based on T_1_ sequences, the system provided the volumes of the following structures: intracranial cavity, tissue categories [grey matter (GM), WM and CSF], subcortical GM structures (putamen, caudate, pallidum, thalamus, hippocampus, amygdala and accumbens).

Based on FLAIR and T_1_ sequences, it is also provided segmentation of WM hyperintensities.

The volBrain system took around 15 min to perform the full analysis.

#### Voxel-based morphometry analysis

Analyses were performed using the T_1_-volume pipeline of the Clinica software platform (www.clinica.run) developed by the ARAMIS Lab (www.aramislab.fr).^44^ This pipeline is a wrapper of different tools of the Statistical Parametric Mapping Software SPM12, London, UK http://www.fil.ion.ucl.ac.uk/spm/).^45^

All T_1_-weighted MRI images were segmented into GM, WM and CSF tissue maps using the SPM unified segmentation routine with the default parameters. A population template was calculated from GM and WM tissue maps using the DARTEL diffeomorphic registration algorithm with the default parameters.^46^ The obtained transformations and a spatial normalization were applied to the GM tissue maps. All maps were modulated to ensure that the overall tissue amount remained constant and normalized to Montreal National Institute space. A 12 mm smoothing was applied as the classification performed better with this parameter than with none or less smoothed images.

#### Cortical thickness analysis

Analyses were performed using the T_1_-freesurfer pipeline of Clinica.^44^ This pipeline is a wrapper of different tools of the FreeSurfer software (http://surfer.nmr.mgh.harvard.edu/).^47^ This processing included segmentation of subcortical structures, extraction of cortical surfaces, cortical thickness (CT) estimation, spatial normalization onto the FreeSurfer surface template (FsAverage) and parcellation of cortical regions.

### Statistical analysis

The difference between groups on demographic and neuropsychological data was evaluated with Mann–Whitney test for non-parametric continuous data, *t*-test for parametric continuous data and *χ*^2^ test for binary data.

### Automated Volumetry Software

The correlation between regions of interest (ROI) volumes (absolute and relative volumes) demographical and neuropsychological data were calculated with a Mann–Whitney test.

All statistical analyses were computed through JMP®, Version 14, SAS Institute Inc., Cary, NC, USA, 1989–2019.

### VBM correlations

Analyses were performed using the statistics-volume pipeline of Clinica. Statistics were corrected for multiple comparisons using the family-wise error (FWE) correction at the peak level with a statistical threshold of *P* < 0.05 FWE.

### CT correlations

Analyses were performed using the statistics-surface command of Clinica. More precisely, a point-wise, vertex-to-vertex model based on the MATLAB SurfStat toolbox (http://www.math.mcgill.ca/keith/surfstat/) was used to conduct a group comparison of whole-brain CT. The data were smoothed using a Gaussian kernel with a full width at half maximum set to 8 mm. The general linear model was used to control for the effect of age, sex and intracranial volume (ICV). Statistics were corrected for multiple comparisons using the random field theory for non-isotropic images A statistical threshold of *P* < 0.05 was first applied (height threshold). An extent threshold of *P* < 0.05 corrected for multiple comparisons was then applied at the cluster level.

### Data availability

All data that support the findings of this study are available on request from the corresponding author, A.M.

## Results

### Neuropsychological tasks

Twenty-eight DM1 patients (13 men and 15 women) performed neuropsychological testing. The mean age was 46.65 years old (±11.17) and the mean educational level of 12.38 years (±3.07). The patients had a mean number of 537.81 CTG triplets (±301.52) and a mean disease duration of 15.25 years (±7.41).

**Figure 1:**
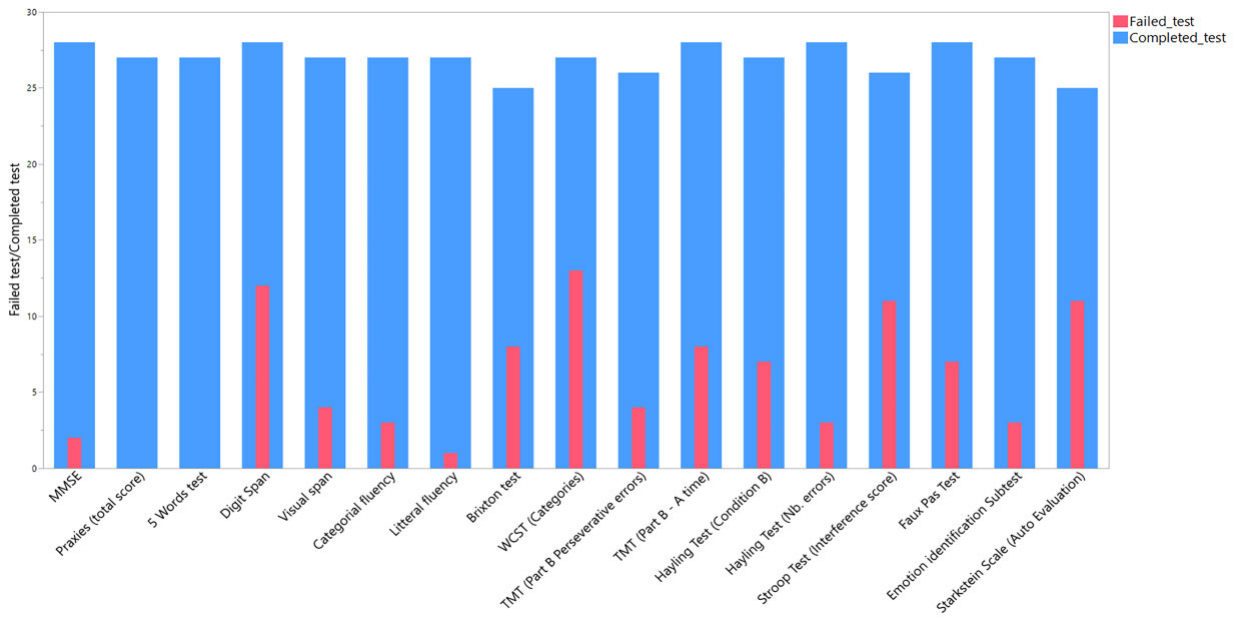
**Neuropsychological evaluation**. Nested in the column: patients with abnormal results on neuropsychological test/28 patients. Entire column: number of patients tested.

Further detailed demographic, clinical and biological data of the DM1 patients are summarized in Supplementary Table 1. Detailed neuropsychological results are illustrated in Supplementary Table 1 and graphically in Fig. 1.

DM1 patients had pathological scores on executive tests, showing difficulties in auditory working memory, inhibition, contextualization, mental flexibility and shifting, with an important sensitivity to interferences. Overall, 21 out of 28 patients (75%) had at least one pathological score on executive tests. Patients showed difficulties in the ToM test. Memory, praxies and attention were relatively spared.

### Apathy evaluation

Eleven out of 27 patients had pathological score on the Starkstein self-assessment scale.

**Figure 2:**
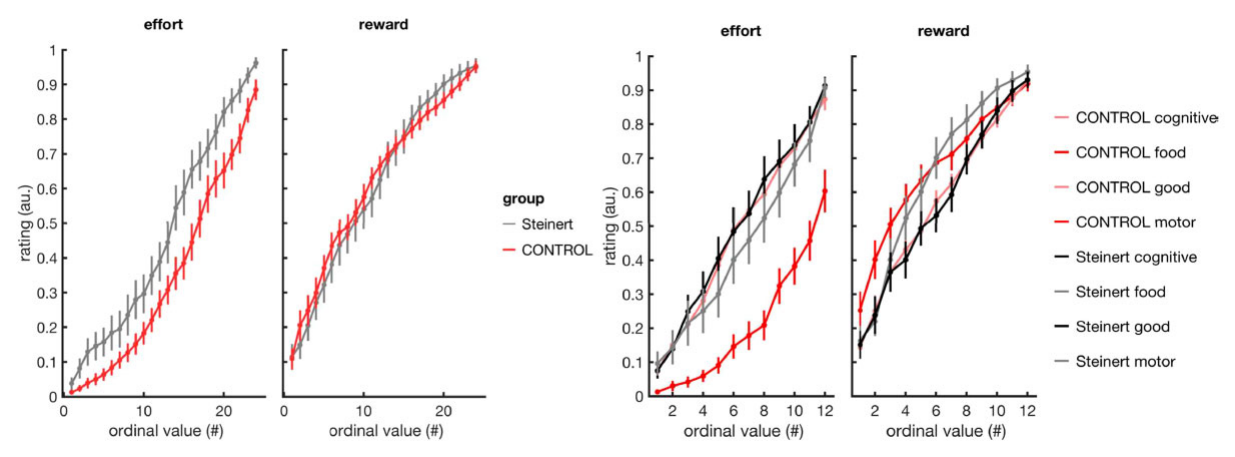
**Perception of reward value and effort cost**. Left: averaged reward value ratings and effort cost ratings in DM1 patients (red) and controls (grey). Items are ordered by rank within each subject. Right: average ratings by subtype of reward (food items versus goods) and subtype of effort (motor versus cognitive effort).

Twenty-two patients and 22 age and sex-matched controls performed the apathy computerized evaluation. DM1 patients and controls gave similar subjective value ratings for rewards (Fig. 2; respectively, 61.5 ± 0.03 and 62.2 ± 0.02, *t* = −0.18, *P* = 0.86, two-tailed unpaired *t*-test). In contrast, DM1 patients perceived efforts as costlier than controls (respectively, 47.1 ± 0.04 and 34.7 ± 0.03, *t* = 2.67, *P* = 0.011). However, this difference was specific to motor efforts (respectively, 44.4 ± 0.04 and 21.1 ± 0.03, *t* = 3.84, *P* < 0.001) and was not found for cognitive efforts (respectively, 50.0± 0.04 and 48.4 ± 0.04, *t* = 0.25, *P* = 0.80), suggesting a reflection of the muscular weakness rather than a general dysfunction in the perception of effort cost. The willingness to exert efforts in order to obtain rewards was similar in both groups (respectively, 52.1 ± 0.04 and 54.7 ± 0.03, *t* = −0.53, *P* = 0.60). Finally, DM1 patients showed no choice impulsivity in inter-temporal choices compared to controls (proportion of patient choices, respectively, 64.4 ± 0.06 and 56.8 ± 0.04, *t* = 1.17, *P* = 0.25); Overall, results did not reveal any alteration in motivational processes in DM1 patients.

### MRI

**Figure 3:**
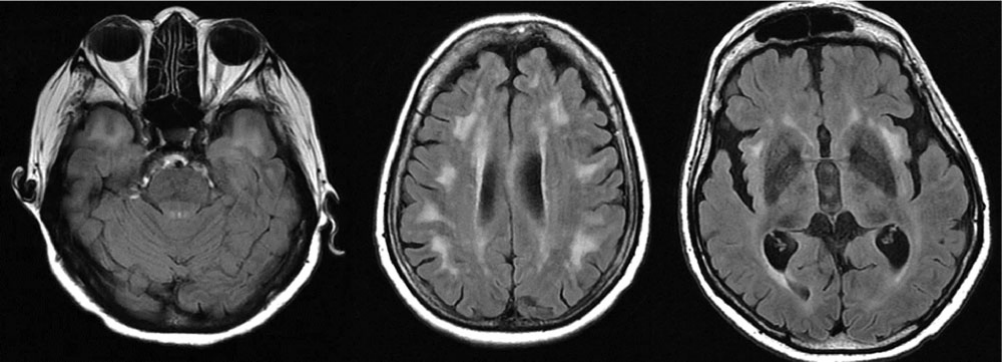
**Examples of diffuse WM intensities involving anterior temporal lobes, periventricular areas and insula**.

Qualitative MRI analysis revealed MRI FLAIR hyperintensities for all patients with specific localizations such as the anterior temporal lobe as displayed in Fig 3.

### Neuroimaging analysis

Clinical and neuroimaging characteristics of the DM1 group and the MRI control group are summarized in Supplementary Table 2 (pX for patients and tX for controls).

No significant differences were reported between the DM1 patients and the MRI control group for age (respectively, 47.09 ± 12.152 and 43.56 ± 12.735) and sex (7 Male and 11 Female and 6 Male and 12 Female). Intracranial volume was significantly lower in DM1 than in the control group (respectively, 1075.781 ± 113.741 cm^3^ and 1400.638 ± 114.078 cm^3^).

### Automated Volumetry Software

Detailed ROI volumes of MRI DM1 Group and MRI Control Group are reported in Supplementary Table 2.

When comparing ROI segmentation in DM1 patients and controls, only a few subcortical structures were significantly smaller in DM1 patients than in controls: Accumbens nucleus (right/left), Thalamus (right/left/total), Caudate (right/left/total) Globus pallidus (right/left) and hippocampus (right/total).

### Voxel-based morphometry analysis

**Figure 4:**
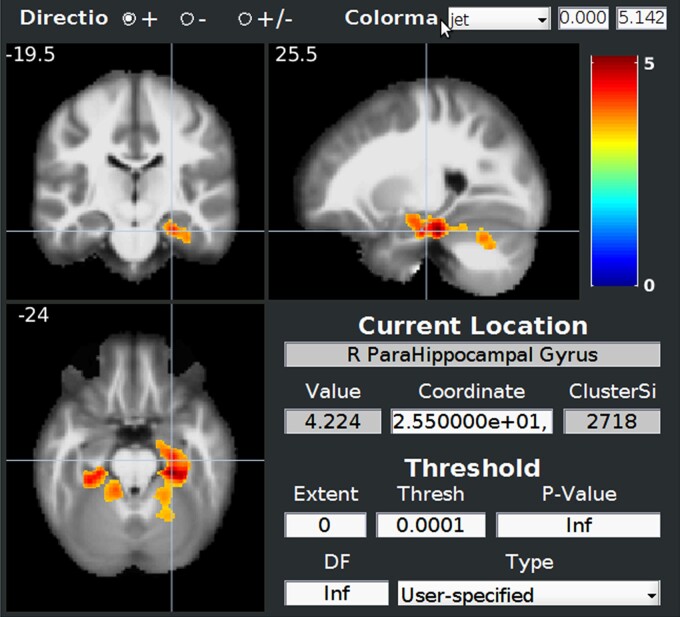
**Comparison of MRI segmentation of GM between 18 DM1 patients and 18 controls showing mostly parahippocampus involvement**. Normalized data were smoothed with an isotropic Gaussian kernel of 8 mm. Statistical analysis was performed using a general linear model with age, sex and total intracranial volume as covariates. Statistics were corrected for multiple comparisons with FWE correction at the cluster level with a height threshold of 0.001. The statistical map is showed through BSPM view.

When comparing ROI segmentation in DM1 patients and controls on GM segmentation, loss of volume concerned mostly right hippocampus and right parahippocampal gyrus and to a smaller extent left parahippocampal gyrus as illustrated in Fig. 4.

### Cortical thickness analysis

**Figure 5:**
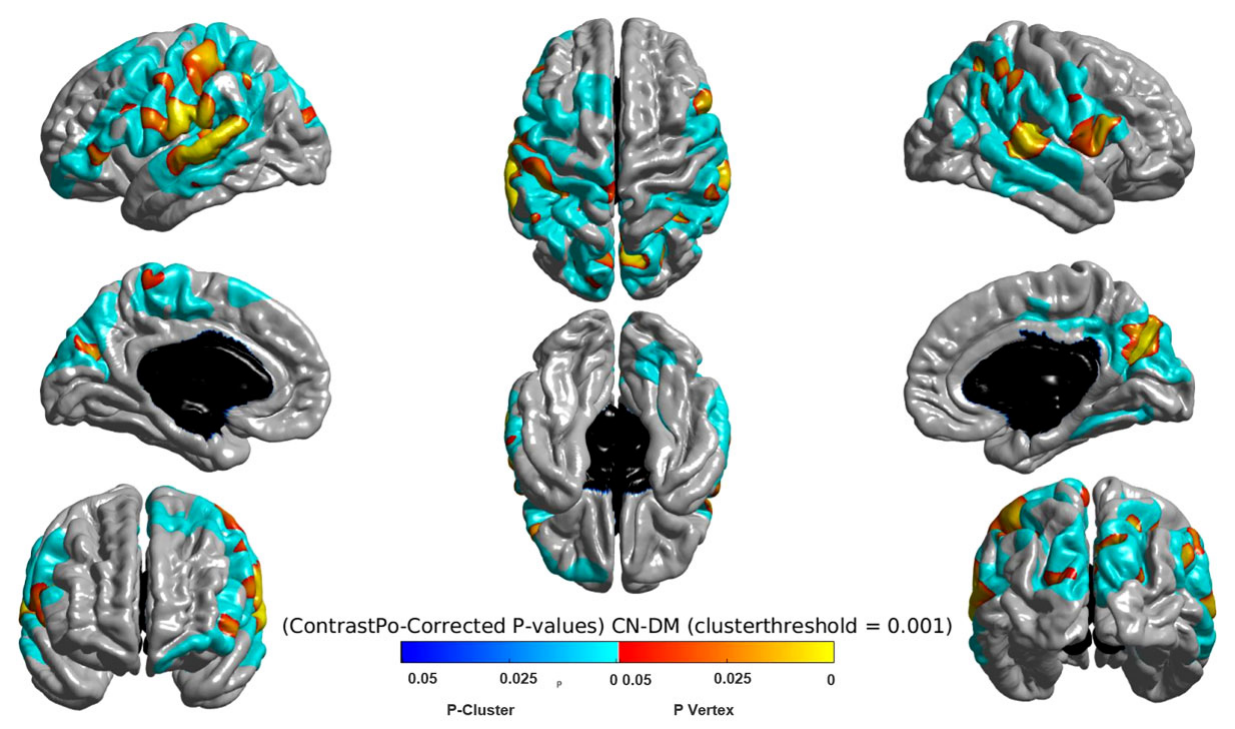
**Comparison of MRI cortical thickness between 18 DM1 patients and 18 controls showing mostly parahippocampal involvement**.

When comparing ROI CT in DM1 patients and controls, the loss of volume concerned mostly the left parahippocampal gyrus and to a smaller extent the right parahippocampal gyrus and the dorsolateral prefrontal cortex (Fig. 5).

### Automated Volumetry Software correlations

#### VBM correlations

**Figure 6:**
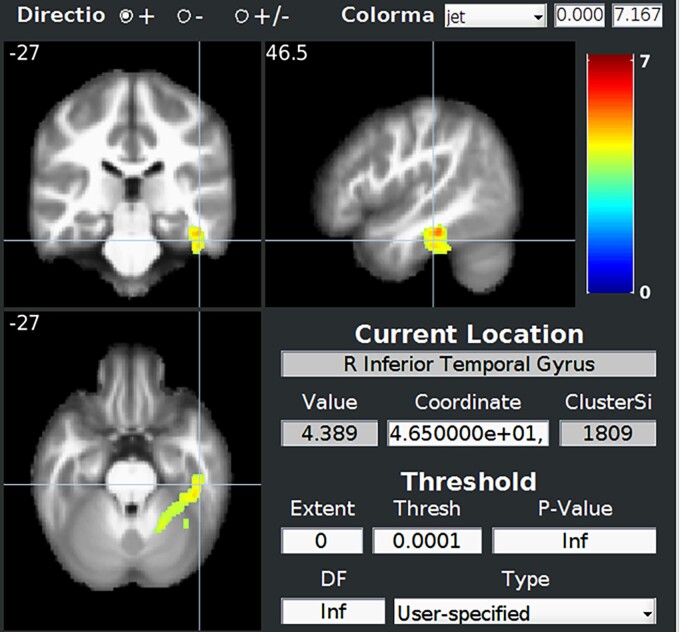
**Correlation between SEA score (social cognition) and GM atrophy showing mostly right parahippocampal gyrus**. Normalized data were smoothed with an isotropic Gaussian kernel of 8 mm. Statistical analysis was performed using a general linear model with age, sex and total intracranial volume as covariates. Statistics were corrected for multiple comparisons with FWE correction at the cluster level with a height threshold of 0.001. The statistical map is showed through BSPM view.

Only the SEA score—global social cognition test—(Fig. 6) and Faux-Pas Score—ToM test—(Supplementary Fig. 1) were correlated with specific voxels when adjusted for multiple corrections. Low SEA score was correlated with right parahippocampal gyrus atrophy and low Faux-Pas Score with right inferior temporal gyrus atrophy.

#### CT correlations

Neuropsychological scores and CT were not statistically correlated.

## Discussion

We report additional data regarding social dysfunction and cognitive disorders in 28 DM1 patients based on specific neuropsychological tests and advanced neuroimaging technologies allowing precise anatomo-clinical correlations.

DM1 patients showed only limited deficits in most of the cognitive functions, as global cognition, episodic memory and visuospatial functions were preserved. Their daily difficulties seemed to rely on a more constrained cognitive disorder, such as frontal dysfunction, according to our hypothese and to the literature.^29,48^ We confirmed the deficit in frontal executive functions. More precisely, mental flexibility, elaboration of rules and contextualization were significantly impaired, with low scores in Brixton and Wisconsin tests. Inhibition was also impaired, with low scores in the Stroop Test as already described in the literature.^12^

Concerning social cognition, only 3 among 28 patients had impaired facial emotion recognition. Controversary results have been described regarding facial emotion recognition but the concerned studies were all based on small samples.^15,17,49^ Seven patients had impaired ToM as explored in the Faux-Pas Test.

These opposite results concerning two different social cognition tests can be explained by the dichotomy between cognitive and affective ToM.

- Affective ToM, related to emotion processing, is explored by the emotion recognition test, and is preserved in our DM1 patients.
- Cognitive ToM, partially related to executive functions,^50^ is explored by the Faux-Pas Test and is more altered in our DM1 patients.

Interestingly, another study also noticed some deficit in ToM, especially concerning tests demanding an high level of executive function, probably related to the cognitive side of the ToM.^15,51^

Thus, social dysfunction in DM1 patients might differ from a unique emotional processing impairment as previously described in the literature^14,17,19^ but more to a cognitive ToM deficit, partially driven by the executive dysfunction, such as contextualization and mental flexibility.

Further investigations on ToM could include complementary tests concerning cognitive ToM,^52^ or through other modalities based on videos for instance.^53^

Concerning motivation and apathy, besides a high score on Starkstein’s Scale (reporting subjective apathy), DM1 patients did not show any deficit in motivation elementary processes. Apathy in DM1 might be caused by an overestimation of the cost of motor efforts, which seems obvious regarding their muscle disease.

Various neuroimaging findings did not point out any frontal lobe atrophy as we first hypothetized.

Regarding subcortical structures, Volbrain segmentation revealed some basal ganglia atrophy.

Interestingly, neuroimaging analysis pointed out damages in the parahippocampal regions in DM1 patients compared to controls [based on Automated Volumetry Software (AVS), VBM and CT] and in the hippocampus (based on AVS that contain patch-based algorithms for hippocampal segmentation^43^ and on VBM) both highly connected regions. Hippocampal atrophy might not have been detected by SPM, due to its inherent performance, lower than Volbrain for hippocampal segmentation.^54^

Regarding hippocampus, there are controversary findings as Weber *et al*.^29^ found hippocampal atrophy^55^ whereas Langbehn *et al*. found hippocampal hypertrophy but with different sample size and imaging techniques.

Regarding the brain injury, two main hypotheses might explain cortical atrophy in DM1 such as an axonal injury leading to cortical atrophy, or a WM disruption leading to axonal degeneration.

The first mechanism is supported by the evolution of GM atrophy through time, as in neurodegenerative disorders,^56^ with Tau deposits involvement.^5,6,57^ Interestingly, neuropathological findings suggest tau aggregation, neurofibrillary tangles mostly in the hippocampus and the parahippocampus,^5,6^ both altered regions in our DM1 patients.

The second mechanism is supported by WM abnormalities on FLAIR-MRI which might be of developmental mechanism as observed in congenital DM1,^58^ implying for instance glia through wrong guidance of axons.^59^

Interestingly, WM disease in our patients was mostly described in the anterior temporal lobes, a region known to be highly connected with the hippocampus and the parahippocampus.^60^

Unfortunately, we could not correlate the WM disease with neuropsychological scores, mostly because the AVS did not provide WM load by region of interest but only through global scores. A more precise segmentation could address this issue and strengthen the length between WM disease and cognition as described previously.^17,49^

Specific cognitive functions are impaired in our patients alongside structural alteration of brain regions of interest (ROI), suggesting neuroanatomical correlations.

Previous VBM and CT studies revealed widely spread GM atrophy with no clear explanation in terms of anatomy clinical relationship.^23,24,27,61^ In our study, all MRIs were performed on a standardized 3 T-MRI protocol with various analysing techniques (VBM, CT and AVS), with age, sex and ICV-corrected statistical analysis. Thus, it might have led to enlightening regional specificities, from which we could hypothesize some neuroanatomical correlations.

Executive dysfunction can be related to the basal ganglia damages, as demonstrated by Volbrain segmentation. Basal ganlia are a key structure for executive functioning (including contextualization) in humans,^62–64^ This basal ganglia alteration is consistent with previous findings in DM1.^10,65^

The absence of frontal lobe atrophy in our study could explain why emotion processing and affective ToM is not impaired as affective ToM is mostly related to fronto-temporal pathways.^66^ This relative preservation of the frontal lobe also supports the fact that motivation processes are maintained as motivation processes are mostly related to the mesial part of the frontal lobe.^42^

Hippocampal and parahippocampal involvement could explain some social dysfunction in DM1 patients through a deficit in the social space navigation.

The concept of social space navigation has been recently proposed as a way to organize our experiences and guide behaviour across all domains of cognition including social cognition.^67,68^ Maintaining a flexible map of our social relations (based on social cues) allows us to adapt to various social contexts by navigating in our social space, and determine our accurate position. This type of navigation is constantly updating our social structure through acquired social cognition knowledge. The hippocampus plays a key role in social navigation by constructing an abstract geometric representation of social relationships during social interactions based on power and affiliation.^67–69^ Hippocampal dysfunction may contribute to maladaptive social behaviour in previously unexpected ways such as the social brain in psychiatric disorders for instance.^69^

Hence, we postulate that WM disruption and GM degeneration lead to 2-fold mechanism responsible for specific social dysfunction in DM1:

- Executive dysfunction (mental flexibility and contextualization), due to basal ganglia.
- Social navigation impairment due to hippocampus and parahippocampus lesions.

Our study has some limitations:

Neuropsychological tests used in this study are not designed for DM1 patients. Nevertheless, previous studies used the same design to evaluate social cognition as no test is available for that purpose (p1)^19,49,51^. We did not include controls for the neuropsychological test. We addressed this issue by using normative data for all tests.

## Conclusion

According to our results, social dysfunction in DM1 patients may result from a 2-fold mechanism causing social dysfunction:

- On one hand, frontal and basal ganglia loops lesion leading to a dysexecutive syndrome, mostly impairing abstraction, mental flexibility, rules understanding, contextualization and cognitive ToM.
- On the other hand, hippocampus and parahippocampus lesions altering the social navigation and emotional contextualization.

Thus, there is a need to evaluate cognition beyond standard neuropsychological assessment to focus on executive function, affective ToM and social navigation with new experimental tasks.

Understanding these cognitive and behavioural disorders might strengthen the patients’ professional situation and educates their caregivers. Interestingly, recent studies revealed positive results of therapy on cognition and it might then be a future direction to improve behavioural disorders in DM1 patients.^70^

## Supplementary Material

fcac111_Supplementary_Data

## Abbreviations

AVS =: Automated Volumetry Software
CT =: cortical thickness
DM1 =: myotonic dystrophy type 1
GM =: grey matter
ROI =: regions of interest
SEA =: Social Emotion Assessment
SPM =: Statistical Parametric Mapping Software
ToM =: Theory of Mind
VBM =: Voxel-based morphometry
WM =: white matter
