# Supplementary material for: Unravelling the impact of frontal lobe impairment for social dysfunction in myotonic dystrophy type 1
Source: Brain Commun. 2022 May 17;4(3):fcac111. doi: 10.1093/braincomms/fcac111 (PMC9123843; doi:10.1093/braincomms/fcac111)
Supplement: fcac111_Supplementary_Data [file fcac111_supplementary_data.zip › Supplementary Table 1.pdf]

Label	Mean (± SD)
Age	46.65 (± 11.17)
Sex	13 Male (46.43%). 15 Female (53.57%)
Educational Level (Years)	12.38 (± 3.07)
Laterality	24 Right-Handed (88.89%). 3 Left-handed (11.11%). 1 ND
Transmission	15 Paternal (75%). 5 Maternal (25%). 8 ND
CTG Triplets (Nb)	537.81 (± 301.52)
Disease Duration (Years)	15.25 (± 7.41)
MDRS Score	2.63 (± 1.08)
Walton Score	1.85 (± 1.26)
Cardiac failure	17 Yes (60.71%). 11 No (39.28%)
Ocular failure	14 Yes (50%). 14 No (50%)
Hypersomnia	15 Yes (53.57%). 13 No (46.43%)
Digestive symptoms	22 Yes (78.57%). 6 No (21.42%)
Respiratory failure	19 Yes (67.86%). 9 No (32.14%). 1 ND
Endocrinological failure	25 Yes (89.29%). 3 No (10.71%)
MMSE	27.71 (± 1.72)
5 Words Total Score	9.52 (± 0.8)
Praxies (symbolic)	5 (± 0)
Praxies (Imitation)	9.81 (± 0.56)
Praxies (abstract)	7.74 (± 0.53)
Digit Span	5.89 (± 2.56)
Direct Digit span	4.89 (± 1.03)
Indirect digit span	3.61 (± 0.74)
Visual span	7.85 (± 2.52)
Direct visual span	5.19 (± 1.21)
Indirect visual span	4.7 (± 0.87)
FAB tot	16.48 (± 1.37)
TMT (Part A time) (Control condition)	36.46 (± 10.76)
TMT (Part A errors) (Control condition)	0.07 (± 0.27)
TMT (Part B time)	109.93 (± 62.44)
TMT (Part B erros)	0.92 (± 1.44)
TMT (Part B Perseverative erros)	0.31 (± 0.84)
TMT (Part B - A time)	73.46 (± 56.4)
TMT (Part A time) (Z Score)	0.04 (± 0.68)
TMT (Part B time) (Z Score)	-1.1 (± 2.42)
TMT Total (Z Score)	-1.5 (± 2.93)
TMT (Part B erros) (Z Score)	-0.62 (± 2.51)
TMT (Part B - A time) (Z Score)	-1.3 (± 2.57)
Categorial fluence	30.89 (± 9.89)
Litteral fluence	21.19 (± 5.68)
Categorial fluence (Z Score)	-0.16 (± 1.23)
Litteral fluence (Z Score)	-0.25 (± 0.86)
WCST Score	14.81 (± 5.03)
WCST (Categories)	4.74 (± 1.48)
WCST (Errors)	10 (± 7.52)
WCST (Perseverative errors)	2.81 (± 3.11)
WCST Cat. Z Score	-3.47 (± 4.83)
WCST Err Z Score	-1.98 (± 2.25)
WCST Pers Z Score	-1.81 (± 2.63)
WCST % ERR PERS	23.06 (± 19.16)
WCST %	19.61 (± 27.54)
Hayling Test (Condition A)	10.8 (± 8.41)
Hayling Test (Condition B)	84.15 (± 40.56)
Hayling Test (Nb. errors)	6.43 (± 4.33)
Hayling A Z Score	-0.96 (± 3.21)
Hayling B Z Score	-1.39 (± 1.73)
Hayling ERR Z Score	-0.33 (± 0.95)
Stroop Test (Denomination score)	68.31 (± 12.65)
Stroop Test (Reading score) (control condition)	49.96 (± 9.23)
Stroop Test (Interference score)	142.35 (± 44.86)
Stroop Test (Denomination score) (Z Score)	-0.86 (± 1.22)
Stroop Test (Reading score) (Z Score)	-1.14 (± 1.17)
Stroop Test (Z Score)	-1.37 (± 1.52)
Brixton Test (Total Score)	17.08 (± 6.38)
Brixton Test (Z Score)	-1.08 (± 1.28)
SEA Emotion Subtets (Total)	28.46 (± 2.76)
SEA Emotion Subtest (Joy)	4.96 (± 0.19)
SEA Emotion Subtest (Surprise)	4.3 (± 0.78)
SEA Emotion subtest (Disgust)	3.78 (± 0.97)
SEA Emotion Subtest (Fear)	3.19 (± 1.04)
SEA Emotion Subtest (Anger)	3.63 (± 1.04)
SEA Emotion Subtest (Sadness)	3.96 (± 1.02)
SEA Emotion Subtest (Neutral)	4.81 (± 0.48)
SEA Emotion Subtets (Total) (Z Score)	-0.29 (± 1.09)
SEA Emotion Subtest (Joy) (Z Score)	0.11 (± 0.11)
SEA Emotion Subtest (Surprise) (Z Score)	-0.55 (± 1.26)
SEA Emotion subtest (Disgust) (Z Score)	-1.01 (± 1.5)
SEA Emotion Subtest (Fear) (Z Score)	0.37 (± 0.95)
SEA Emotion Subtest (Anger) (Z score)	-0.13 (± 1.11)
SEA Emotion Subtest (Sadness) (Z Score)	0.23 (± 0.84)
SEA Emotion Subtest (Neutral) (Z Score)	0.12 (± 1.13)
Faux Pas Test (Total Score)	22.57 (± 6.34)
Faux Pas Test (Control condition)	9.43 (± 1.07)
Faux Pas Test (Total Score) (Z Score)	-0.67 (± 1.76)
Faux Pas Test (Control condition) (Z Score)	-0.19 (± 1.19)
Starkstein Scale (Auto Evaluation)	12.92 (± 4.19)
Mini SEA (Total Score)	24.2 (± 3.19)
