# Supplementary material for: Unravelling the impact of frontal lobe impairment for social dysfunction in myotonic dystrophy type 1
Source: Brain Commun. 2022 May 17;4(3):fcac111. doi: 10.1093/braincomms/fcac111 (PMC9123843; doi:10.1093/braincomms/fcac111)
Supplement: fcac111_Supplementary_Data [file fcac111_supplementary_data.zip › Supplementary Table 2.pdf]

Id	Sex	Age	Tissue WM (cm3)	Tissue WM (%)	Tissue GM (cm3)	Tissue GM (%)	Tissue CSF (cm3)	Tissue CSF (%)
**P001SAFR**	1,00	52.88	425.1597	28.885	726.4983	49.3577	320.2474	21.7573
**P002AUSO**	2,00	53.7	332.0868	28.184	697.8733	59.228	148.3232	12.5881
**P003BOFR**	1,00	52.7	516.5495	35.5794	604.77	41.6559	330.5024	22.7647
**P004MANI**	1,00	39.7	396.8411	28.6123	685.271	49.4082	304.8472	21.9795
**P005BRCE**	2,00	38.6	379.3666	31.227	588.6219	48.4515	246.8788	20.3215
**P007GIFR**	1,00	61.3	408.0172	27.3741	615.6567	41.3048	466.8471	31.3211
**P009JOST**	2,00	39.9	422.635	36.2275	585.7399	50.2086	158.2378	13.5639
**P010ROMA**	2,00	67.5	402.8167	33.5588	558.0901	46.4946	239.4256	19.9466
**P014RIGR**	1,00	24.9	616.9869	39.9337	717.6686	46.4502	210.3739	13.6162
**P015OKST**	2,00	32.5	408.025	29.9026	698.2379	51.1711	258.2521	18.9263
**P018GOCH**	2,00	53.2	518.3349	33.5357	619.7165	40.095	407.5707	26.3694
**P019CHCE**	2,00	66.1	480.5901	36.3051	544.0243	41.0971	299.139	22.5978
**P020VOMA**	2,00	39.6	463.8996	34.5712	697.8335	52.0046	180.1356	13.4242
**P024DEEL**	2,00	52.8	415.8602	36.8152	560.5102	49.6208	153.2179	13.564
**P025LAMI**	1,00	30.0	503.6791	34.519	755.0668	51.7475	200.3906	13.7335
**P026LATH**	1,00	55.3	496.5544	33.8732	619.7891	42.2798	349.5785	23.847
**P027PRVA**	2,00	46.4	343.4576	33.9703	507.8517	50.2299	159.7445	15.7998
**P028GAJE**	2,00	39.8	468.0416	37.6686	581.9434	46.8356	192.5385	15.4958
**T002AR**	1,00	###	465.4756	33.2846	685.8226	49.0408	247.1745	17.6746
**T003AD**	2,00	###	502.2254	38.9807	653.7493	50.7414	132.4204	10.2779
**T005BP**	2,00	###	609.2391	45.1684	655.3271	48.5853	84.2502	6.2462
**T006CE**	1,00	###	598.4559	39.1309	719.8045	47.0654	211.1092	13.8037
**T008CT**	2,00	###	408.8738	33.9469	584.5228	48.5302	211.0553	17.5229
**T009CV**	1,00	###	620.4582	38.8242	807.9551	50.5565	169.7104	10.6194
**T010DA**	2,00	###	495.3832	35.9697	729.7453	52.9867	152.0954	11.0436
**T011DM**	2,00	###	403.6708	35.7506	595.8637	52.7719	129.5958	11.4775
**T012DP**	1,00	###	529.2627	37.1749	692.4012	48.6336	202.047	14.1916
**T013DT**	2,00	###	518.4239	36.9129	730.3095	51.9996	155.7175	11.0874
**T014GC**	2,00	###	516.8354	36.0929	732.3468	51.1431	182.7751	12.764
**T018LE**	2,00	###	615.4949	41.1924	739.6402	49.5009	139.0609	9.3067
**T019MP**	2,00	###	480.2184	34.6487	713.8907	51.5087	191.8533	13.8426
**T021PV**	1,00	###	529.1629	35.1684	839.7658	55.8112	135.7269	9.0205
**T022RC**	1,00	###	511.9174	34.3398	737.9574	49.5028	240.8647	16.1574
**T025TL**	2,00	###	542.2042	38.602	697.382	49.6498	165.015	11.7482
**T026VT**	2,00	###	517.8535	38.7978	650.3306	48.723	166.5669	12.4792
**T029SE**	2,00	###	490.0215	33.5058	743.2806	50.8226	229.1968	15.6716

Tissue Brain (cm3)	Tissue Brain %	Tissue IC (cm3)	Tissue IC (%)	Brainstem (cm3)	Brainstem (%)	Lat. ventricles Tot. (cm3)
1151.6579	78.2427	1471.9053	100,00	23.5638	1.6009	58.9989
1029.9602	87.4119	1178.2834	100,00	19.3119	1.639	19.466
1121.3194	77.2353	1451.8218	100,00	22.5457	1.5529	32.2308
1082.1122	78.0205	1386.9594	100,00	21.8458	1.5751	33.4239
967.9885	79.6785	1214.8673	100,00	16.8104	1.3837	32.3381
1023.6739	68.6789	1490.521	100,00	21.0784	1.4142	43.9189
1008.3749	86.4361	1166.6127	100,00	18.2716	1.5662	7.8027
960.9069	80.0534	1200.3325	100,00	20.6812	1.723	13.8531
1334.6554	86.3838	1545.0293	100,00	27.2556	1.7641	9.0183
1106.2628	81.0737	1364.515	100,00	20.7595	1.5214	11.8769
1138.0514	73.6306	1545.6221	100,00	22.0267	1.4251	58.4801
1024.6144	77.4022	1323.7534	100,00	23.859	1.8024	24.8139
1161.7331	86.5758	1341.8687	100,00	22.5164	1.678	6.9285
976.3704	86.436	1129.5884	100,00	19.1378	1.6942	8.7266
1258.7459	86.2665	1459.1365	100,00	24.7605	1.6969	25.2536
1116.3435	76.153	1465.922	100,00	23.8404	1.6263	38.168
851.3092	84.2002	1011.0538	100,00	15.5199	1.535	10.8511
1049.9851	84.5042	1242.5236	100,00	22.3407	1.798	9.7524
1151.2982	82.3254	1398.4727	100,00	26.5549	1.8989	24.9946
1155.9747	89.7221	1288.3951	100,00	21.182	1.6441	4.5562
1264.5662	93.7538	1348.8164	100,00	22.7905	1.6897	6.8278
1318.2604	86.1963	1529.3696	100,00	27.2588	1.7824	10.3806
993.3967	82.4771	1204.452	100,00	18.7123	1.5536	8.2283
1428.4133	89.3806	1598.1237	100,00	24.5184	1.5342	3.7037
1225.1284	88.9564	1377.2239	100,00	23.8909	1.7347	15.5916
999.5345	88.5225	1129.1303	100,00	22.0231	1.9504	10.1466
1221.6639	85.8084	1423.7108	100,00	25.194	1.7696	14.7898
1248.7334	88.9126	1404.4509	100,00	24.5607	1.7488	11.5319
1249.1823	87.236	1431.9573	100,00	24.3027	1.6972	6.6595
1355.1351	90.6933	1494.196	100,00	28.0243	1.8755	17.0557
1194.1091	86.1574	1385.9624	100,00	22.2369	1.6044	87.11
1368.9287	90.9795	1504.6556	100,00	28.1243	1.8691	9.41
1249.8748	83.8426	1490.7395	100,00	22.1441	1.4854	22.9791
1239.5862	88.2518	1404.6012	100,00	24.063	1.7132	12.9251
1168.1841	87.5208	1334.751	100,00	24.4759	1.8337	10.5451
1233.3022	84.3284	1462.4989	100,00	22.9805	1.5713	9.1539

Lat. ventricles Tot. (%)	Lat. ventricles R (cm3)	Lat. ventricles R (%)	Lat. ventricles L (cm3)	Lat. ventricles L (%)	Lat. ventricles Asym.	Caudate Tot. (cm3)
4.01	29.4693	2,00	29.5296	2.01	-0.20468	7.1365
1.65	9.8335	0.83	9.6326	0.82	2.0639	3.9159
2.22	15.5796	1.07	16.6512	1.15	-6.6498	5.136
2.41	18.9451	1.37	14.4788	1.04	26.7252	7.1946
2.66	19.3309	1.59	13.0072	1.07	39.1095	6.9518
2.95	18.8512	1.26	25.0676	1.68	-28.3086	5.815
0.67	2.807	0.24	4.9957	0.43	-56.1012	5.4634
1.15	5.679	0.47	8.1741	0.68	-36.0216	5.9743
0.58	6.0514	0.39	2.9669	0.19	68.4055	8.3607
0.87	5.0994	0.37	6.7775	0.5	-28.2575	6.0923
3.78	33.6971	2.18	24.783	1.6	30.486	5.9821
1.87	14.3002	1.08	10.5136	0.79	30.5199	7.302
0.52	3.7304	0.28	31.98	0.24	15.3679	7.9496
0.77	3.5868	0.32	5.1398	0.46	-35.5916	5.4425
1.73	10.956	0.75	14.2976	0.98	-26.4645	9.2895
2.6	13.9114	0.95	24.2567	1.65	-54.2093	6.9191
1.07	4.783	0.47	6.0681	0.6	-23.6861	4.847
0.78	5.5744	0.45	4.1779	0.34	28.6384	5.9341
1.79	12.8745	0.92	12.1201	0.87	6.0363	7.3989
0.35	2.448	0.19	2.1082	0.16	14.9151	6.531
0.51	2.9703	0.22	3.8575	0.29	-25.9875	8.5167
0.68	4.6347	0.3	5.7459	0.38	-21.4077	7.485
0.68	4.4497	0.37	3.7786	0.31	16.3106	6.0247
0.23	1.9566	0.12	1.7471	0.11	11.3153	8.3977
1.13	7.7733	0.56	7.8183	0.57	-0.57789	8.2177
0.9	4.9667	0.44	5.1799	0.46	-4.2013	6.328
1.04	6.1175	0.43	8.6722	0.61	-34.5469	6.4312
0.82	5.2613	0.37	6.2706	0.45	-17.5051	8.294
0.47	3.2413	0.23	3.4182	0.24	-53.14	8.6764
1.14	6.7302	0.45	10.3254	0.69	-42.1583	8.1091
0.63	2.7709	0.2	5.9401	0.43	-72.7632	6.5533
0.63	4.7183	0.31	4.6917	0.31	0.56628	7.0266
1.54	12.5901	0.84	10.389	0.7	19.1575	7.2166
0.92	6.1094	0.43	6.8157	0.49	-10.9289	7.7308
0.79	4.4682	0.33	6.0769	0.46	-30.5099	6.418
0.63	4.6893	0.32	4.4647	0.31	4.9077	8.8174

Caudate Tot. (%)	Caudate R(cm3)	Caudate R(%)	Caudate L (cm3)	Caudate L (%)	Caudate Asym.	Putamen Tot. (cm3)
0.48	3.5288	0.24	3.6077	0.25	-2.2092	8.8682
0.33	2.0325	0.17	1.8834	0.16	7.6131	14.7684
0.35	2.6439	0.18	2.4921	0.17	5.9132	6.9574
0.52	3.5091	0.25	3.6856	0.27	-4.9073	8.1262
0.57	3.4099	0.28	3.5419	0.29	-3.7985	6.4119
0.39	2.9569	0.2	2.8581	0.19	3.3999	6.831
0.47	2.7375	0.23	2.7258	0.23	0.42796	6.6629
0.5	3.036	0.25	2.9383	0.24	3.272	7.7587
0.54	4.2735	0.28	4.0872	0.26	4.4563	8.4336
0.45	3.101	0.23	2.9913	0.22	3.6027	6.076
0.39	2.9533	0.19	3.0288	0.2	-2.5219	6.8314
0.55	3.4991	0.26	3.803	0.29	-8.3231	6.7256
0.59	3.9966	0.3	3.953	0.29	1.0972	9.0963
0.48	2.8156	0.25	2.6268	0.23	6.9384	6.0732
0.64	4.7706	0.33	4.5189	0.31	5.4182	8.3788
0.47	3.3124	0.23	3.6067	0.25	-8.5076	7.5907
0.48	2.3871	0.24	2.4599	0.24	-3.0041	5.4575
0.48	3.0094	0.24	2.9247	0.24	2.8552	7.2243
0.53	3.8137	0.27	3.5852	0.26	6.1781	8.4162
0.51	3.3276	0.26	3.2034	0.25	3.8015	8.0909
0.63	4.307	0.32	4.2097	0.31	2.2841	9.5689
0.49	3.6692	0.24	3.8157	0.25	-3.9136	8.639
0.5	3.0685	0.25	2.9562	0.25	3.727	7.0582
0.53	4.2386	0.27	4.1591	0.26	1.8951	10.1262
0.6	4.1333	0.3	4.0844	0.3	1.1894	7.8084
0.56	3.1947	0.28	3.1333	0.28	1.9387	7.1446
0.45	3.2793	0.23	3.1519	0.22	3.964	7.9067
0.59	4.1618	0.3	4.1322	0.29	0.71254	7.7014
0.61	4.2513	0.3	4.4251	0.31	-4.0055	8.8438
0.54	4.028	0.27	4.081	0.27	-1.3072	8.1476
0.47	3.2523	0.23	3.301	0.24	-1.4852	7.6105
0.47	3.5274	0.23	3.4992	0.23	0.80432	9.3874
0.48	3.5857	0.24	3.6309	0.24	-1.252	8.5434
0.55	3.8318	0.27	3.899	0.28	-1.7402	8.642
0.48	3.272	0.25	3.146	0.24	3.9251	7.3645
0.6	4.4313	0.3	4.3861	0.3	1.0262	8.0396

Putamen Tot. (%)	Putamen R (cm3)	Putamen R (%)	Putamen L (cm3)	Putamen L (%)	Putamen Asym.	Thalamus Tot. (cm3)
0.6	4.5603	0.31	4.3079	0.29	5.6927	10.4129
1.25	7.4683	0.63	7.3001	0.62	2.2785	13.5132
0.48	3.5068	0.24	3.4506	0.24	1.6142	9.7223
0.59	4.1522	0.3	3.974	0.29	4.3855	9.3221
0.53	3.0722	0.25	3.3397	0.27	-8.3451	9.3736
0.46	3.526	0.24	3.305	0.22	6.4678	8.7642
0.57	3.3052	0.28	3.3578	0.29	-1.5791	10.7072
0.65	3.9577	0.33	3.8009	0.32	4.0416	9.5818
0.55	4.2366	0.27	4.1971	0.27	0.93648	12.8917
0.45	3.0328	0.22	3.0432	0.22	-0.34171	12.3729
0.44	3.4441	0.22	3.3873	0.22	1.6628	10.1796
0.51	3.3166	0.25	3.409	0.26	-2.7464	9.1492
0.68	4.4944	0.33	4.6019	0.34	-2.3642	11.0356
0.54	3.0558	0.27	3.0174	0.27	1.263	8.7496
0.57	4.0895	0.28	4.2893	0.29	-4.7682	11.6599
0.52	3.8132	0.26	3.7775	0.26	0.94259	9.7981
0.54	2.7296	0.27	2.7279	0.27	0.060637	7.8595
0.58	3.628	0.29	3.5964	0.29	0.87483	10.8059
0.6	4.1805	0.3	4.2357	0.3	-1.3135	10.506
0.63	3.9929	0.31	4.0979	0.32	-2.5951	11.4135
0.71	4.7528	0.35	4.8161	0.36	-1.3245	12.8583
0.56	4.3166	0.28	4.3225	0.28	-0.13641	12.4884
0.59	3.5349	0.29	3.5233	0.29	0.32721	10.1292
0.63	5.0662	0.32	5.06	0.32	0.12224	14.0775
0.57	3.9302	0.29	3.8782	0.28	1.3299	12.8289
0.63	3.5472	0.31	3.5974	0.32	-1.4049	10.0078
0.56	3.9066	0.27	4.0001	0.28	-2.3644	11.7785
0.55	3.8834	0.28	3.8181	0.27	1.6963	11.6921
0.62	4.414	0.31	4.4298	0.31	-0.35887	12.0303
0.55	4.032	0.27	4.1156	0.28	-2.0501	11.9484
0.55	3.9553	0.29	3.6551	0.26	7.8898	11.0342
0.62	4.6771	0.31	4.7103	0.31	-0.70526	12.6032
0.57	4.3135	0.29	4.2299	0.28	1.9585	11.9384
0.62	4.337	0.31	4.305	0.31	0.74215	13.263
0.55	3.6931	0.28	3.6715	0.28	0.5864	10.5861
0.55	3.863	0.26	4.1765	0.29	-7.7994	12.9987

Thalamus Tot. (%)	Thalamus R (cm3)	Thalamus R (%)	Thalamus L (cm3)	Thalamus L (%)	Thalamus Asym.	Globus Pallidus Tot. (cm3)
0.71	5.0735	0.34	5.3394	0.36	-5.1059	2.7523
1.15	6.6021	0.56	6.9111	0.59	-4.5732	1.9602
0.67	4.8869	0.34	4.8355	0.33	1.0575	1.7463
0.67	4.405	0.32	4.9172	0.35	-10.9886	2.328
0.77	4.5523	0.37	4.8212	0.4	-5.738	2.0173
0.59	4.4964	0.3	4.2678	0.29	5.2177	2.4652
0.92	5.2016	0.45	5.5056	0.47	-5.6775	2.0653
0.8	4.7946	0.4	4.7872	0.4	0.15584	1.8455
0.83	6.5278	0.42	6.3639	0.41	2.5438	2.6587
0.91	5.9892	0.44	6.3837	0.47	-6.3766	1.7544
0.66	4.9128	0.32	5.2668	0.34	-6.9567	1.9621
0.69	4.5716	0.35	4.5776	0.35	-0.13025	2.6731
0.82	5.3659	0.4	5.6697	0.42	-5.5056	2.1551
0.77	4.2913	0.38	4.4583	0.39	-3.8168	1.5996
0.8	5.7505	0.39	5.9094	0.4	-2.7249	2.5945
0.67	4.9572	0.34	4.8409	0.33	2.3732	2.4538
0.78	3.911	0.39	3.9485	0.39	-0.95439	1.6883
0.87	5.2987	0.43	5.5072	0.44	-3.8576	1.8718
0.75	5.3456	0.38	5.1604	0.37	3.5262	2.6829
0.89	5.6748	0.44	5.7387	0.45	-1.1187	2.0912
0.95	6.3636	0.47	6.4947	0.48	-2.0401	2.5864
0.82	6.2737	0.41	6.2147	0.41	0.94365	2.6372
0.84	5.0508	0.42	5.0784	0.42	-0.54468	1.8309
0.88	6.7921	0.43	7.2854	0.46	-7.0092	2.6197
0.93	6.356	0.46	6.4729	0.47	-1.8213	2.1197
0.89	5.0739	0.45	4.9339	0.44	2.7984	2.137
0.83	5.9816	0.42	5.7969	0.41	3.135	2.1491
0.83	5.9005	0.42	5.7916	0.41	1.8622	2.2341
0.84	6.035	0.42	5.9953	0.42	0.65954	2.324
0.8	5.9521	0.4	5.9963	0.4	-0.7393	2.8387
0.8	5.6646	0.41	5.3696	0.39	5.3467	1.9855
0.84	6.1668	0.41	6.4364	0.43	-4.2793	2.0685
0.8	5.7977	0.39	6.1407	0.41	-5.7463	2.5977
0.94	6.5193	0.46	6.7437	0.48	-3.385	2.6187
0.79	5.3672	0.4	5.2189	0.39	2.8012	2.2168
0.89	6.3894	0.44	6.6093	0.45	-3.3828	2.3018

Globus Pallidus Tot. (%)	Globus Pallidus R (cm3)	Globus Pallidus R (%)	Globus Pallidus L (cm3)	Globus Pallidus L (%)	Globus Pallidus Asym.	Hippocamp. Tot. (cm3)
0.19	1.4642	0.1	1.2881	0.09	12.7971	7.3101
0.17	1.0012	0.08	0.95897	0.08	4.3081	8.8598
0.12	0.86918	0.06	0.87709	0.06	-0.9058	7.6336
0.17	1.1959	0.09	1.1321	0.08	5.4824	8.3467
0.17	1.0271	0.08	0.99024	0.08	3.6514	7.9504
0.17	1.2404	0.08	1.2249	0.08	1.2552	8.7934
0.18	1.0216	0.09	1.0437	0.09	-2.1384	7.2332
0.15	0.95295	0.08	0.89254	0.07	6.5465	7.7743
0.17	1.2723	0.08	1.3864	0.09	-8.589	7.4266
0.13	0.87944	0.06	0.87499	0.06	0.50719	7.7525
0.13	0.98504	0.06	0.97705	0.06	0.81411	8.687
0.2	1.3101	0.1	1.363	0.1	-3.9565	7.5702
0.16	1.0708	0.08	1.0843	0.08	-1.2561	8.5594
0.14	0.75052	0.07	0.84906	0.08	-12.3202	7.317
0.18	1.3204	0.09	1.274	0.09	3.5768	7.1669
0.17	1.2375	0.08	1.2163	0.08	1.723	8.1062
0.17	0.83063	0.08	0.85765	0.08	-3.2016	5.9506
0.15	0.8606	0.07	1.0112	0.08	-1.6092	8.1374
0.19	1.2638	0.09	1.4191	0.1	-11.5813	8.526
0.16	1.0924	0.08	0.99877	0.08	8.9552	7.2645
0.19	1.3006	0.1	1.2858	0.1	1.1396	7.1719
0.17	1.3359	0.09	1.3014	0.09	2.6173	8.091
0.15	0.92381	0.08	0.90713	0.08	1.822	6.942
0.16	1.3554	0.08	1.2643	0.08	6.9524	7.9715
0.15	1.0835	0.08	1.0362	0.08	4.4669	7.2158
0.19	1.0298	0.09	1.1072	0.1	-7.2485	6.499
0.15	1.0869	0.08	1.0622	0.07	2.3005	8.201
0.16	1.1135	0.08	1.1205	0.08	-0.62652	9.07
0.16	1.1291	0.08	1.1949	0.08	-5.6675	7.8004
0.19	1.3708	0.09	1.4679	0.1	-6.8458	8.2745
0.14	1.0085	0.07	0.97704	0.07	3.1674	7.8328
0.14	1.0843	0.07	0.9842	0.07	9.6799	8.3402
0.17	1.3486	0.09	1.2491	0.08	7.6651	7.5872
0.19	1.3586	0.1	1.2601	0.09	7.5269	7.5744
0.17	1.0818	0.08	1.1351	0.09	-4.8052	7.841
0.16	1.189	0.08	1.1128	0.08	6.6207	8.6587

Hippocamp. Tot. (%)	Hippocamp. R (cm3)	Hippocamp. R (%)	Hippocamp. L (cm3)	Hippocamp. L (%)	Hippocamp. Asym.	Amygdala Tot. (cm3)
0.5	3.6437	0.25	3.6664	0.25	-0.61948	1.4332
0.75	4.0867	0.35	4.7731	0.41	-15.4957	0.58152
0.53	3.8603	0.27	3.7733	0.26	2.2793	1.6055
0.6	4.2119	0.3	4.1348	0.3	1.8469	1.6468
0.65	3.9978	0.33	3.9526	0.33	1.1363	1.6177
0.59	4.3572	0.29	4.4362	0.3	-1.7986	1.6959
0.62	3.8293	0.33	3.4039	0.29	11.7626	1.5919
0.65	3.9543	0.33	3.82	0.32	3.4573	1.5319
0.48	3.7035	0.24	3.7232	0.24	-0.53173	1.832
0.57	3.9775	0.29	3.7751	0.28	5.2224	1.4067
0.56	4.3839	0.28	4.3031	0.28	1.8592	1.5335
0.57	3.7613	0.28	3.8089	0.29	-1.2593	1.5827
0.64	4.2417	0.32	4.3177	0.32	-1.7746	1.652
0.65	3.673	0.33	3.6441	0.32	0.79026	1.3689
0.49	3.7396	0.26	3.4273	0.23	8.7128	2.482
0.55	4.0498	0.28	4.0563	0.28	-0.16048	1.8123
0.59	2.9458	0.29	3.0048	0.3	-1.9835	1.4153
0.65	4.0744	0.33	4.063	0.33	0.28092	1.4818
0.61	4.2985	0.31	4.2275	0.3	1.6645	1.6268
0.56	3.5347	0.27	3.7298	0.29	-5.3706	1.5762
0.53	3.4728	0.26	3.6991	0.27	-6.3084	1.711
0.53	4.1011	0.27	3.99	0.26	2.7466	1.4781
0.58	3.5329	0.29	3.4091	0.28	3.5671	1.6776
0.5	4.1228	0.26	3.8487	0.24	6.8767	1.7524
0.52	3.5621	0.26	3.6537	0.27	-2.5397	1.576
0.58	3.1804	0.28	3.3186	0.29	-4.2521	1.1283
0.58	4.014	0.28	4.187	0.29	-4.22	1.5149
0.65	4.4852	0.32	4.5848	0.33	-2.1948	1.6143
0.54	3.8538	0.27	3.9466	0.28	-2.3802	1.7337
0.55	4.1172	0.28	4.1573	0.28	-0.9705	1.6759
0.57	3.9628	0.29	3.87	0.28	2.3705	1.4674
0.55	4.1661	0.28	4.1742	0.28	-0.19361	2.0524
0.51	3.5882	0.24	3.999	0.27	-10.8281	1.9409
0.54	3.9796	0.28	3.5948	0.26	10.1611	1.8639
0.59	3.9212	0.29	3.9198	0.29	0.036717	1.5302
0.59	4.2313	0.29	4.4274	0.3	-4.5284	1.5708

Amygdala Tot. (%)	Amygdala R(cm3)	Amygdala R(%)	Amygdala L (cm3)	Amygdala L (%)	Amygdala Asym.	Accumbens Tot. (cm3)
0.1	0.67172	0.05	0.76145	0.05	-12.5219	0.70191
0.05	0.30004	0.03	0.28149	0.02	6.3806	0.072291
0.11	0.85257	0.06	0.75292	0.05	12.4138	0.53464
0.12	0.88348	0.06	0.76331	0.06	14.5949	0.68043
0.13	0.78246	0.06	0.83528	0.07	-6.5292	0.51423
0.11	0.8398	0.06	0.85613	0.06	-19.26	0.49081
0.14	0.7956	0.07	0.79625	0.07	-0.081599	0.58582
0.13	0.73711	0.06	0.7948	0.07	-7.5321	0.55928
0.12	0.922	0.06	0.90998	0.06	1.3121	0.88337
0.1	0.70518	0.05	0.70147	0.05	0.52715	0.57616
0.1	0.76141	0.05	0.77206	0.05	-1.3889	0.57327
0.12	0.8461	0.06	0.73661	0.06	13.8353	0.537
0.12	0.86474	0.06	0.78729	0.06	9.3764	0.7903
0.12	0.65081	0.06	0.71807	0.06	-9.8276	0.44725
0.17	1.3417	0.09	1.1403	0.08	16.2231	0.73847
0.12	0.92444	0.06	0.88786	0.06	4.0377	0.48946
0.14	0.74679	0.07	0.66847	0.07	11.0678	0.45889
0.12	0.72949	0.06	0.75235	0.06	-3.0853	0.52443
0.12	0.78874	0.06	0.83803	0.06	-6.0606	0.76932
0.12	0.80866	0.06	0.76752	0.06	5.2205	0.81434
0.13	0.85697	0.06	0.85403	0.06	0.34453	0.92182
0.1	0.73318	0.05	0.74496	0.05	-1.5945	0.70624
0.14	0.80705	0.07	0.87056	0.07	-7.5717	0.59855
0.11	0.90449	0.06	0.8479	0.05	6.4581	0.93809
0.11	0.79794	0.06	0.77809	0.06	2.5194	0.7254
0.1	0.588	0.05	0.54029	0.05	8.4569	0.52294
0.11	0.73852	0.05	0.77638	0.05	-4.9975	0.61569
0.11	0.78538	0.06	0.82893	0.06	-5.395	0.6773
0.12	0.89501	0.06	0.83868	0.06	6.4989	0.88073
0.11	0.82954	0.06	0.8464	0.06	-2.0125	0.64966
0.11	0.76666	0.06	0.70078	0.05	8.9796	0.53382
0.14	0.99954	0.07	1.0528	0.07	-5.1928	0.72987
0.13	0.94787	0.06	0.99305	0.07	-4.6552	0.7153
0.13	0.90417	0.06	0.9597	0.07	-5.9589	0.77355
0.11	0.78309	0.06	0.7471	0.06	4.7037	0.55565
0.11	0.81277	0.06	0.758	0.05	6.9732	1.0128

Accumbens Tot. (%)	Accumbens R(cm3)	Accumbens R(%)	Accumbens L (cm3)	Accumbens L (%)	Accumbens Asym.	Tot. lesions
0.05	0.36898	0.03	0.33292	0.02	10.2748	33,00
0.01	0.038384	**0,00**	0.033906	**0,00**	12.3894	52,00
0.04	0.24438	0.02	0.29025	0.02	-17.1598	13,00
0.05	0.30582	0.02	0.37461	0.03	-20.2192	12,00
0.04	0.24391	0.02	0.27032	0.02	-10.2703	16,00
0.03	0.24842	0.02	0.2424	0.02	2.4518	22,00
0.05	0.27667	0.02	0.30915	0.03	-11.0865	39,00
0.05	0.24842	0.02	0.31086	0.03	-22.3301	19,00
0.06	0.40777	0.03	0.47559	0.03	-15.3547	15,00
0.04	0.29735	0.02	0.27881	0.02	6.435	9,00
0.04	0.27066	0.02	0.30261	0.02	-11.1455	19,00
0.04	0.2294	0.02	0.3076	0.02	-29.1262	28,00
0.06	0.37823	0.03	0.41207	0.03	-8.5633	8,00
0.04	0.19353	0.02	0.25372	0.02	-26.9129	7,00
0.05	0.34918	0.02	0.38929	0.03	-10.8626	6,00
0.03	0.20001	0.01	0.28945	0.02	-36.5449	23,00
0.05	0.23275	0.02	0.22613	0.02	2.8846	13,00
0.04	0.26423	0.02	0.2602	0.02	1.5385	8,00
0.06	0.35553	0.03	0.41379	0.03	-15.1456	14,00
0.06	0.4171	0.03	0.39724	0.03	4.878	
0.07	0.43401	0.03	0.4878	0.04	-11.6707	13,00
0.05	0.32071	0.02	0.38553	0.03	-18.3552	9,00
0.05	0.28035	0.02	0.3182	0.03	-12.6474	5,00
0.06	0.40759	0.03	0.53049	0.03	-26.2017	7,00
0.05	0.37721	0.03	0.34819	0.03	8,00	15,00
0.05	0.27758	0.02	0.24536	0.02	12.3223	8,00
0.04	0.30437	0.02	0.31132	0.02	-2.2585	8,00
0.05	0.26439	0.02	0.41291	0.03	-43.8576	1,00
0.06	0.43323	0.03	0.44751	0.03	-3.2432	8,00
0.04	0.32764	0.02	0.32202	0.02	1.7305	2,00
0.04	0.23808	0.02	0.29573	0.02	-21.5989	
0.05	0.33748	0.02	0.39239	0.03	-15.0442	6,00
0.05	0.34217	0.02	0.37313	0.03	-8.655	25,00
0.06	0.37543	0.03	0.39812	0.03	-5.8645	4,00
0.04	0.25047	0.02	0.30518	0.02	-19.6891	23,00
0.07	0.49687	0.03	0.51592	0.04	-3.7618	

Tot. lesion Vol. (cm3)	Tot. lesion Vol. (%)	Tot. lesion burden	Periv. lesions	Periv. lesion Vol. (cm3)	Periv. lesion Vol. (%)	Periv. lesion burden
23.9969	1.6312	5.4179	9,00	22.8462	1553,00	5.1581
30.9241	2.6898	8.3474	5,00	21.4163	1.8628	5.781
4.3678	0.30128	0.8785	6,00	3.7021	0.25537	0.74461
2.2688	0.16358	0.5794	8,00	2.1792	0.15712	0.55652
5.8086	0.47928	1.5122	7,00	5.3535	0.44173	1.3938
39.2536	2.6318	9.5245	2,00	38.6252	2.5897	9.372
22.97	0.19735	0.52256	10,00	1.7165	0.14747	0.39048
1.5136	0.12603	0.36932	7,00	1.31	0.10908	0.31965
1.6243	0.10498	0.25756	10,00	1.5539	0.10042	0.24638
43.77	0.32072	10.03	6,00	3.8287	0.28054	0.87733
35.5771	2.2985	6.7623	6,00	35.3588	2.2844	6.7209
18.5552	1.4033	3.7954	4,00	17.1249	1.2951	3.5028
0.51138	0.03814	0.10134	5,00	0.29361	0.021898	0.058184
0.3717	0.033275	0.089831	5,00	0.36405	0.03259	0.08798
0.99557	0.069097	0.19791	5,00	9.94	0.068988	0.1976
8.0203	0.5493	1.5863	10,00	7.6275	0.5224	1.5086
5.3974	0.53279	1.5472	6,00	4.8513	0.47889	1.3907
0.35263	0.028605	0.082153	5,00	0.03634	0.0029478	0.0084661
0.058998	0.004212	0.012563	4,00	0.03286	0.0023459	0.0069969
0.21634	0.016038	0.035282	7,00	0.17219	0.012765	0.028082
0.9667	0.06316	0.15452	7,00	0.65036	0.042492	0.10395
0.08143	0.0067598	0.019712	4,00	0.079507	0.0066001	0.019247
0.32363	0.020209	0.053723	4,00	0.30948	0.019326	0.051375
0.30233	0.021972	0.061361	12,00	0.29772	0.021637	0.060424
0.055156	0.00489	0.013405	3,00	0.011775	0.0010439	0.0028618
0.48915	0.034401	0.093663	5,00	0.22915	0.016115	0.043877
0.019448	0.0013864	0.0037876	0.0023459	0.0023459	0.0023459	0.0023459
0.73472	0.050651	0.13946	4,00	0.72047	0.049668	0.13676
0.020642	0.0013836	0.0033333	2,00	0.020642	0.0013836	0.0033333
0.071841	0.0047724	0.013394	3,00	0.066191	0.004397	0.012341
0.21337	0.014309	0.039635	8,00	0.15061	0.0101	0.027978
0.11494	0.0081829	0.021392	3,00	0.11181	0.0079603	0.02081
8.0203	0.5493	1.5863	10,00	7.6275	0.5224	1.5086

Juxtacort. lesions	Juxtacort. lesion Vol. (cm3)	Juxtacort. lesion Vol. (%)	Juxtacort. lesion burden	Deep white lesions	Deep white lesion Vol. (cm3)	Deep white lesion Vol. (%)
18,00	1.0962	0.074516	0.2475	6,00	0.054476	0.003703
43,00	9.4638	0.82318	2.5546	4,00	0.044018	0.0038287
2,00	0.63208	0.0436	0.12713	5,00	0.033604	0.002318
2,00	0.075489	0.0054427	0.019278	2,00	0.014102	0.0010168
5,00	0.43147	0.035602	0.11233	4,00	0.023623	0.0019492
14,00	0.56236	0.037704	0.13645	6,00	0.066008	0.0044257
8,00	0.28315	0.024327	0.064416	21,00	0.29744	0.025555
9,00	0.11903	0.0099108	0.029044	3,00	0.084541	0.0070389
3,00	0.037815	0.0024439	0.005996	2,00	0.032659	0.0021107
1,00	0.54388	0.039852	0.12463	2,00	0.0044519	0.00032621
5,00	0.12687	0.0081965	0.024115	8,00	0.09138	0.0059038
15,00	1.2515	0.094648	0.25599	9,00	0.17879	0.013521
2,00	0.01652	0.0012321	0.0032738	1,00	0.20125	0.01501
1,00	0.0070689	0.00063281	0.0017084	1,00	0.00058907	0.00005273
0.0023459	0.0023459	0.0023459	0.0023459	1,00	0.0015703	0.00010899
3,00	0.16182	0.011083	0.032006	10,00	0.23094	0.015817
2,00	0.45987	0.045395	0.13183	5,00	0.086226	0.0085116
2,00	0.31562	0.025602	0.07353	1,00	0.00067296	0.00005458
8,00	0.023898	0.0017061	0.0050886	2,00	0.0022404	0.00015995
2,00	0.013245	0.00098194	0.0021601	4,00	0.030906	0.0022912
1,00	0.31466	0.020559	0.050295	1,00	0.0016827	0.00010994
1,00	0.0019235	0.00015968	0.00046565	0.0023459	0.0023459	0.0023459
2,00	0.0053054	0.0003313	0.00088071	1,00	0.0088423	0.00055216
3,00	0.0046157	0.00033545	0.0009368	0.0023459	0.0023459	0.0023459
5,00	0.043381	0.0038461	0.010544	0.0023459	0.0023459	0.0023459
2,00	0.25924	0.018231	0.049638	1,00	0.00077153	0.00005426
1,00	0.019448	0.0013864	0.0037876	0.0023459	0.0023459	0.0023459
1,00	0.0015835	0.00010916	0.00030056	3,00	0.012668	0.00087329
0.0023459	0.0023459	0.0023459	0.0023459	0.0023459	0.0023459	0.0023459
3,00	0.0056504	0.00037535	0.0010535	0.0023459	0.0023459	0.0023459
14,00	0.05104	0.0034229	0.0094814	3,00	0.011714	0.00078558
0.0023459	0.0023459	0.0023459	0.0023459	1,00	0.0031276	0.00022267
3,00	0.16182	0.011083	0.032006	10,00	0.23094	0.015817

Deep white lesion burden
0.012299
0.011882
0.0067589
0.0036014
0.0061503
0.016016
0.067666
0.020628
0.0051784
0.0010201
0.017369
0.03657
0.039881
0.00014236
0.00031216
0.045677
0.024717
0.00015678
0.00047706
0.0050403
0.00026896
0.0023459
0.0014679
0.0023459
0.0023459
0.00014773
0.0023459
0.0024045
0.0023459
0.0023459
0.0021761
0.00058209
0.045677
