# Supplementary figures and images for: Unravelling the impact of frontal lobe impairment for social dysfunction in myotonic dystrophy type 1
Source: Brain Commun. 2022 May 17;4(3):fcac111. doi: 10.1093/braincomms/fcac111 (PMC9123843; doi:10.1093/braincomms/fcac111)

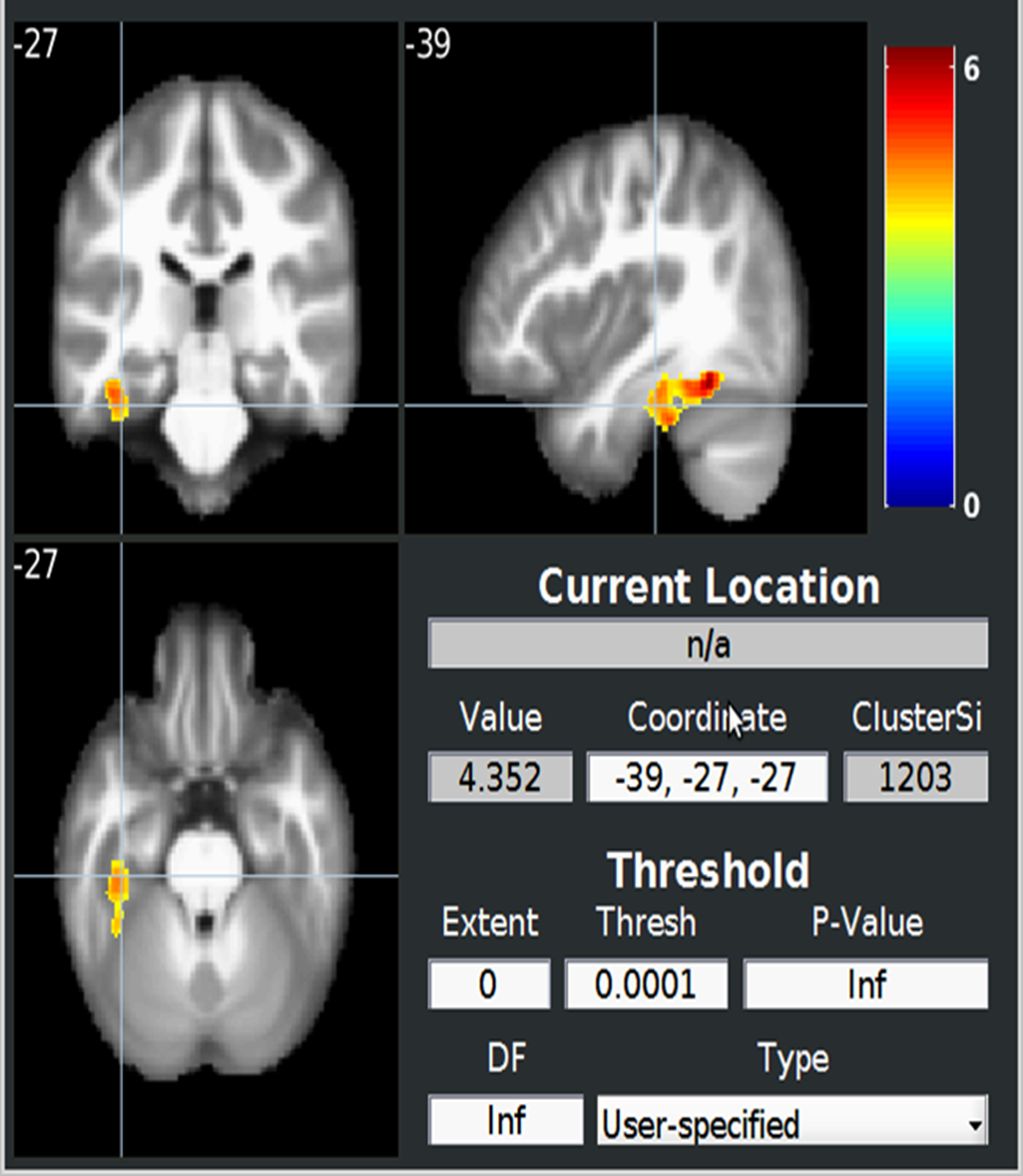

Supplement: fcac111_Supplementary_Data [file fcac111_supplementary_data.zip › Supplementary Figure.tif]
